# Identification of a bacteriocin and its cognate immunity factor expressed by *Moraxella catarrhalis*

**DOI:** 10.1186/1471-2180-9-207

**Published:** 2009-09-25

**Authors:** Ahmed S Attia, Jennifer L Sedillo, Todd C Hoopman, Wei Liu, Lixia Liu, Chad A Brautigam, Eric J Hansen

**Affiliations:** 1 Department of Microbiology, University of Texas Southwestern Medical Center, Dallas, TX, USA; 2 Department of Internal Medicine, Pulmonary and Critical Care Medicine, University of Texas Southwestern Medical Center, Dallas, TX, 75390, USA; 3 Department of Biochemistry, University of Texas Southwestern Medical Center, Dallas, TX, USA; 4 Department of Microbiology and Immunology, Faculty of Pharmacy, Cairo University, Cairo, Egypt

## Abstract

**Background:**

Bacteriocins are antimicrobial proteins and peptides ribosomally synthesized by some bacteria which can effect both intraspecies and interspecies killing.

**Results:**

*Moraxella catarrhalis *strain E22 containing plasmid pLQ510 was shown to inhibit the growth of *M. catarrhalis *strain O35E. Two genes (*mcbA *and *mcbB*) in pLQ510 encoded proteins predicted to be involved in the secretion of a bacteriocin. Immediately downstream from these two genes, a very short ORF (*mcbC*) encoded a protein which had some homology to double-glycine bacteriocins produced by other bacteria. A second very short ORF (*mcbI*) immediately downstream from *mcbC *encoded a protein which had no significant similarity to other proteins in the databases. Cloning and expression of the *mcbI *gene in *M. catarrhalis *O35E indicated that this gene encoded the cognate immunity factor. Reverse transcriptase-PCR was used to show that the *mcbA*, *mcbB*, *mcbC*, and *mcbI *ORFs were transcriptionally linked. This four-gene cluster was subsequently shown to be present in the chromosome of several *M. catarrhalis *strains including O12E. Inactivation of the *mcbA*, *mcbB*, or *mcbC *ORFs in *M. catarrhalis *O12E eliminated the ability of this strain to inhibit the growth of *M. catarrhalis *O35E. In co-culture experiments involving a *M. catarrhalis *strain containing the *mcbABCI *locus and one which lacked this locus, the former strain became the predominant member of the culture after overnight growth in broth.

**Conclusion:**

This is the first description of a bacteriocin and its cognate immunity factor produced by *M. catarrhalis*. The killing activity of the McbC protein raises the possibility that it might serve to lyse other *M. catarrhalis *strains that lack the *mcbABCI *locus, thereby making their DNA available for lateral gene transfer.

## Background

*Moraxella catarrhalis*, formerly known as both *Neisseria catarrhalis *and *Branhamella catarrhalis *[1], is a gram-negative bacterium that can frequently be isolated from the nasopharynx of healthy persons [2-4]. For many years, *M. catarrhalis *was considered to be a harmless commensal [1-4]. About twenty years ago, it was acknowledged that *M. catarrhalis *was a pathogen of the respiratory tract [5], and since then much evidence has accumulated which indicates that *M. catarrhalis *causes disease in both adults and children. *M. catarrhalis *is one of the three most important causes of otitis media in infants and very young children [3,6]. In adults, this bacterium can cause infectious exacerbations of chronic obstructive pulmonary disease (COPD), and one recent study estimates that, in the United States alone, *M. catarrhalis *may cause 2 million-4 million infectious exacerbations of COPD annually [7].

The ability of *M. catarrhalis *to colonize the mucosa of the upper respiratory tract (i.e., nasopharynx) is undoubtedly linked to its expression of different adhesins for various human cells and antigens [8-15]. In addition, this bacterium clearly has the metabolic capability to survive and grow in this environment in the presence of the normal flora. A recent study [16] identified a number of different metabolic pathways encoded by the *M. catarrhalis *ATCC 43617 genome which could be involved in the colonization process. It is likely that *M. catarrhalis *forms a biofilm in concert with these other bacteria in the nasopharynx [17], although only a few *M. catarrhalis *gene products relevant to biofilm formation have been identified to date [13,18,19]. Similarly, there is little known about what extracellular gene products are synthesized by *M. catarrhalis *and released into the extracellular milieu. A study from Campagnari and colleagues [15] found that one or two very large proteins with some similarity to the filamentous hemagglutinin (FhaB) of *Bordetella pertussis *could be found in *M. catarrhalis *culture supernatant fluid. Using the nucleotide sequence of the genome of *M. catarrhalis *ATCC 43617, Murphy and co-workers [20] identified a large number (i.e., 348) of proteins that had signal sequences, among which may be proteins that are released from the *M. catarrhalis *cell. Another group showed that *M. catarrhalis *culture supernatant fluid contained several different proteins as detected by SDS-PAGE analysis, but the identity of the individual proteins was not determined [21].

In the present study, we report the first identification of a bacteriocin that is produced by *M. catarrhalis*. Bacteriocins are proteins or peptides secreted or released by some bacteria that can effect both intraspecies and interspecies killing, and are responsible for some types of bacterial antagonism [for reviews see [22,23]]. The locus encoding this peptide bacteriocin was identified initially in a *M. catarrhalis *plasmid and subsequently shown to be present in the chromosome of some *M. catarrhalis *strains. Four genes encoding the bacteriocin, relevant secretion factors, and a host immunity factor were shown to form a polycistronic operon (*mcbABCI*). This bacteriocin was shown to be active against *M. catarrhalis *strains lacking this operon. Recombinant methods were used to confirm the identity of the cognate immunity factor which does not resemble other proteins in the databases. In competitive co-culture assays, a *M. catarrhalis *strain expressing this bacteriocin became the predominant member of a mixed culture in which the other strain lacked the *mcbABCI *locus.

## Results

### *M. catarrhalis *strain E22 produces a factor that inhibits the growth of *M. catarrhalis *strain O35E

**Figure 1:**
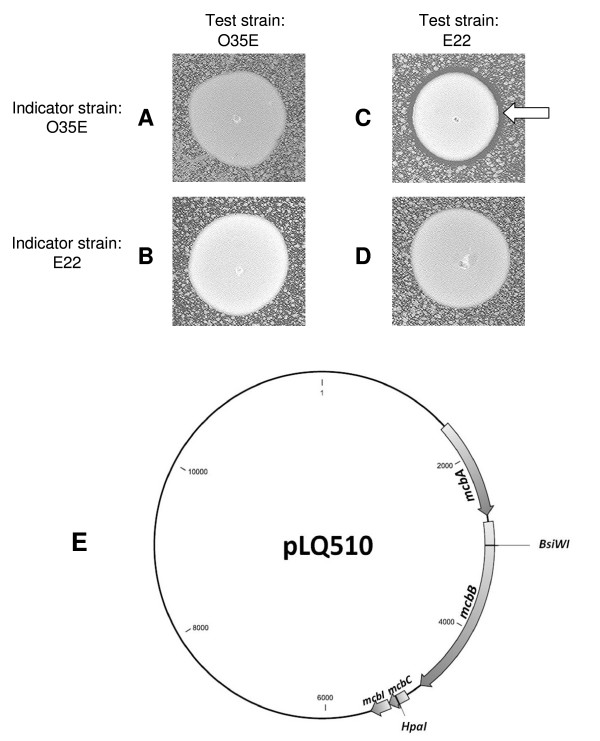
**Killing of *M. catarrhalis *O35E by *M. catarrhalis *E22 carrying pLQ510**. Test strains and indicator strains were grown on BHI agar plates as described in Materials and Methods. Panels: A, O35E test strain on O35E indicator; B, O35E test strain on E22 indicator; C, E22 test strain on O35E indicator; D, E22 test strain on E22 indicator. The white arrow in panel C indicates the zone of killing of the indicator strain by the test strain. Panel E, schematic of pLQ510 indicating the four ORFs located in the *mcb *locus. The nucleotide sequence of pLQ510 is available at GenBank under accession no. AF129811. The positions of the restriction sites used to insert kanamycin resistance cassettes in the *mcbB *and *mcbC *genes are indicated.

Wild-type *M. catarrhalis *strain E22 was originally described as the host for the plasmid pLQ510 [24]. As reported previously [25], two of the ORFs in this plasmid were predicted to encode products with similarity to proteins involved in secretion of bacteriocins in other bacteria. Upon testing the E22 strain in a bacteriocin production assay using wild-type *M. catarrhalis *strain O35E as the indicator strain, the growth of the indicator strain was inhibited in the area immediately around the E22 strain (Figure 1C). In control experiments, O35E did not kill either itself (Figure 1A) or E22 (Figure 1B) and E22 did not kill itself (Figure 1D). This result indicated that strain E22 was capable of producing one or more factors that inhibited the growth of strain O35E.

### Characterization of relevant protein products encoded by pLQ510

In a previous publication [25], ORF1 (now designated as *M. catarrhalis *bacteriocin gene A or *mcbA*) in pLQ510 (Figure 1E) was described as encoding a protein with homology to the colicin V secretion protein of *E. coli *[26] whereas ORF2 (now designated *mcbB*) (Figure 1E) encoded a protein that was most similar to the colicin V secretion ATP-binding protein CvaB [26]. Analysis of the similarities between the amino acid sequences of the McbA and McbB proteins and those of proteins in sequence databases was next assessed using BLAST [blastp and PSI-BLAST [27]]. Both McbA and McbB were found to be members of well-populated protein families. McbA belongs to the HlyD family of so-called membrane-fusion proteins (MFPs). These proteins form a periplasm-spanning tube that extends from an ABC-type transporter in the plasma membrane to a TolC-like protein in the outer membrane [28]. An alignment [29] of McbA to *E. coli *HlyD showed that the two proteins are approximately 19% identical. Likewise, the primary structure of McbB is similar to that of the *E. coli *protein HlyB protein; their sequence identity is ~27%. HlyB is an ABC-type transporter that is presumably dimeric. It has two main domains: the N-terminal domain spans the plasma membrane, facilitating the export of its cognate substrate, while the C-terminal domain uses the energy of ATP hydrolysis to drive the export of the substrate against a concentration gradient [28]. Although the degree of sequence identity between the *M. catarrhalis *and *E. coli *proteins is modest, it is not unreasonable to assume that they may share analogous functions.

### Identification of the *M. catarrhalis *bacteriocin and immunity factor genes

**Figure 2:**
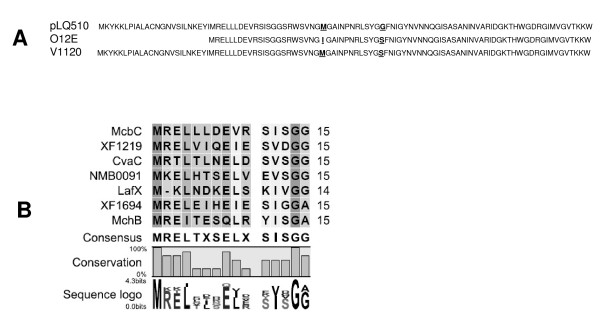
**Putative bacteriocin proteins encoded by the *mcb *locus**. (A) Amino acid sequence of the predicted McbC proteins encoded by the *mcb *locus in plasmid pLQ510, *M. catarrhalis *O12E, and *M. catarrhalis *V1120. Residues that differ among the proteins are underlined and bolded. (B) Alignment of the amino acid sequence of the putative leader of the *M. catarrhalis *O12E McbC protein with that of leader peptides of proven and hypothetical double-glycine peptides from other bacteria including CvaC [GenBank: CAA11514] and MchB [GenBank: CAD56170] of *E. coli*, NMB0091 [GenBank: NP_273152] of *Neisseria meningitidis*, XF1219 [GenBank:AAF84029] and XF1694 [GenBank: AF84503] of *Xylella fastidiosa *and LafX [GenBank: AAS08589] of *Lactobacillus johnsonii*. Highly conserved amino acids are shaded with dark grey. This latter figure is adapted from that published by Michiels et al [30].

Immediately downstream from *mcbB*, two overlapping and small putative ORFs were detected. The first of these, designated *mcbC *(Figure 1E), contained 303-nt in pLQ510 and was predicted to encode a protein containing 101 amino acids (Figure 2A). BLAST analysis showed that this polypeptide had little similarity to other proteins or known bacteriocins. However, examination of the sequence of amino acids 25-39 in this protein revealed that it was similar to the leader sequence of the double-glycine (GG) bacteriocin family including *E. coli *colicin V (CvaC) and other double-glycine peptides of both gram-negative and gram-positive bacteria [30,31] (Figure 2B).

The second very small ORF was designated *mcbI *(Figure 1E) and overlapped the *mcbC *ORF, contained 225 nt, and encoded a predicted protein comprised of 74 amino acids. Similar to McbC, this small protein did not have significant sequence similarity to other proteins in sequence databases. Although the BLAST search algorithm can find similar sequences, they are statistically poor (P ~ 0.925 for McbC; P ~ 0.983 for McbI). Despite this fact, the results of subjecting these sequences to the PSIPRED [32] secondary-structure prediction algorithm suggest that these proteins are not simply random coils. This algorithm predicts that approximately 50% of the residues of both of these small proteins belong to a regular secondary structural element. For McbI, the algorithm predicts four α-helices; the average confidence score for residues with non-coil predictions is 6.13, where 9 = highest confidence and 0 = low confidence. The prediction for McbI is superior to that for McbC. For McbC, the algorithm predicts seven β-strands and one α-helix; the average confidence score for these secondary structural elements is 5.34. It is noteworthy that the PSIPRED algorithm predicts four α-helices for McbI; the colicin E9 immunity factor is known to comprise three α-helices and one 3_10 _helix [33].

### Analysis of potential transcriptional linkage among the ORFs in the *mcb *locus

**Figure 3:**
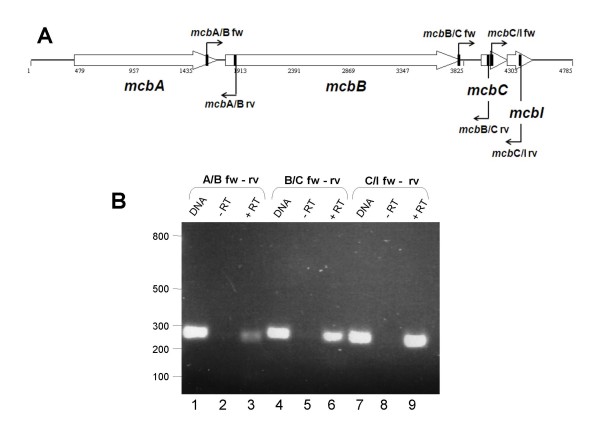
**Reverse transcriptase-PCR analysis of the *mcbABCI *locus in pLQ510**. (A) Schematic drawing showing the three sets of oligonucleotide primers that collectively spanned the three intergenic regions. (B) RT-PCR analysis of possible transcriptional linkage among the ORFs in the *mcbABCI *locus in pLQ510. RT-PCR was carried out as described in Materials and Methods. Lanes 1, 4, and 7 contain PCR products derived from pLQ510 DNA. Lanes 2, 5, and 8 are RT-PCR negative controls in which *M. catarrhalis *E22 RNA was incubated in the absence of reverse transcriptase. Lanes 3, 6, and 9 show the products obtained when these same primer pairs were used in RT-PCR with RNA from *M. catarrhalis *E22. Size markers (in bp) are present on the left side of panel B.

Reverse transcriptase-PCR was used to assess possible linkage among the *mcbA*, *mcbB*, *mcbC*, and *mcbI *ORFs in pLQ510. Primer pairs were designed to overlap the three regions separating these ORFs (Figure 3A). RNA was isolated from *M. catarrhalis *E22 in the logarithmic phase of growth, reverse-transcribed, and then PCR-amplified using these three pairs of oligonucleotide primers. Positive RT-PCR reactions were observed for all three sets of primers (Figure 3B), indicating that these four ORFs are likely transcribed together to yield a polycistronic mRNA in *M. catarrhalis *E22.

### The *mcb *locus is present in the chromosome of some *M. catarrhalis *wild-type strains

**Figure 4:**
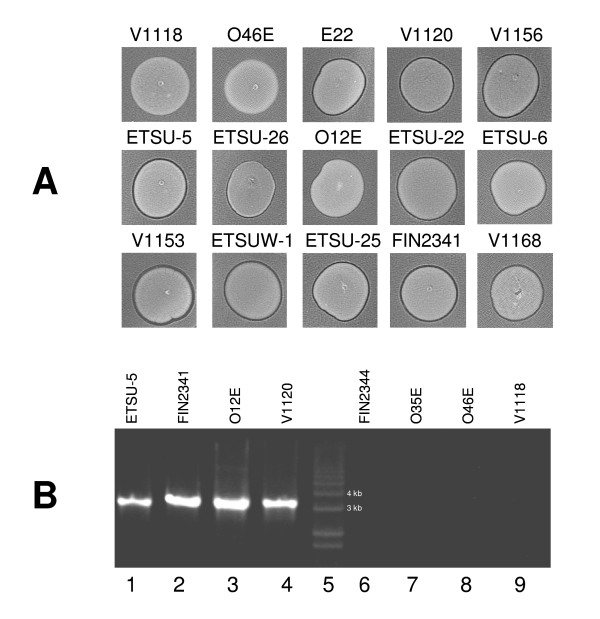
**Detection of bacteriocin production by wild-type *M. catarrhalis *strains**. (A) Fifteen *M. catarrhalis *strains tested for bacteriocin production with strain O35E as the indicator strain. Thirteen of these strains were positive as shown here together with two negative strains (V1118 and O46E). (B) Agarose DNA gel electrophoretic analysis of PCR products obtained from four bacteriocin-positive strains (lane 1, ETSU-5; lane 2, FIN 2341; lane 3, O12E; lane 4, V1120) and 4 bacteriocin-negative strains (lane 6, FIN 2344; lane 7, O35E; lane 8, O46E; lane 9, V1118) with the oligonucleotide primers AA247 (binds within *mcbA*) and pLQ510-rp1 (binds within *mcbC*). Lane 5 contains a set of DNA size markers.

A total of 55 wild-type *M. catarrhalis *strains were tested in the bacteriocin production assay with strain O35E as the indicator strain. Thirteen strains (E22, V1120, V1156, ETSU-5, ETSU-26, O12E, ETSU-22, ETSU-6, V1153, ETSU-W-1, ETSU-25, FIN2341, and V1168) were found to inhibit the growth of O35E (Figure 4A and Table 1). To determine whether the *mcbABCI *locus was present in these strains, chromosomal DNA isolated from four of these putative bacteriocin-producing strains and from four strains that did not inhibit strain O35E was used in PCR with primers that would amplify a 3.2-kb product spanning the *mcbABC *genes as found in pLQ510 (Figure 4B). All four of the bacteriocin-producing strains (Figure 4B, lanes 1-4) yielded the predicted 3.2-kb PCR product whereas the four bacteriocin-negative strains (Figure 4B, lanes 6-9) did not yield any detectable PCR product. Subsequent plasmid DNA extraction from two of these bacteriocin-positive strains (O12E and V1120) showed no plasmid DNA detectable by agarose gel electrophoretic methods (data not shown), suggesting that the *mcbABCI *locus in these strains was located in the chromosome.

**Table 1:** Bacterial strains and plasmids used in this study.

Bacterial Strains	Description	Bacteriocin Production	Source or Reference
*M. catarrhalis*			
O35E	Wild-type strain	**-**	[54]
O35EΔ*mapA*	O35E *mapA *mutant, spectinomycin-resistant	**-**	[34]
E22	Wild-type strain containing plasmid pLQ510	**+**	[24]
O12E	Wild-type strain	**+**	[55]
O12E-Sm^r^	O12E *rpsL *mutant, streptomycin-resistant	**+**	[53]
O12EΔ*mcbA*	O12E with a deletion in the *mcbA *ORF	**-**	This study
O12E.*mcbB::kan*	O12E with a *kan *cartridge inserted in the *mcbB *ORF	**-**	This study
O12EΔ*mcbB*	O12E with a deletion in the *mcbB *ORF	**-**	This study
O12E.*mcbC::kan*	O12E with a *kan *cartridge inserted in the *mcbC *ORF	**-**	This study
O12EΔ*mcbC*	O12E with a deletion in the *mcbC *ORF	**-**	This study
V1069	Wild-type strain	**-**	Frederick Henderson
V1118	Wild-type strain	**-**	Frederick Henderson
V1120	Wild-type strain	**+**	Frederick Henderson
V1122	Wild-type strain	**-**	Frederick Henderson
V1126	Wild-type strain	**-**	Frederick Henderson
V1127	Wild-type strain	**-**	Frederick Henderson
V1129	Wild-type strain	**-**	Frederick Henderson
V1130	Wild-type strain	**-**	Frederick Henderson
V1145	Wild-type strain	**-**	Frederick Henderson
V1153	Wild-type strain	**+**	Frederick Henderson
V1156	Wild-type strain	**+**	Frederick Henderson
V1166	Wild-type strain	**-**	Frederick Henderson
V1168	Wild-type strain	**+**	Frederick Henderson
V1171	Wild-type strain	**-**	Frederick Henderson
ETSU-1	Wild-type strain	**-**	Steven Berk
ETSU-4	Wild-type strain	**-**	Steven Berk
ETSU-5	Wild-type strain	**+**	Steven Berk
ETSU-6	Wild-type strain	**+**	Steven Berk
ETSU-9	Wild-type strain	**-**	Steven Berk
ETSU-12	Wild-type strain	**-**	Steven Berk
ETSU-13	Wild-type strain	**-**	Steven Berk
ETSU-15	Wild-type strain	**-**	Steven Berk
ETSU-17	Wild-type strain	**-**	Steven Berk
ETSU-19	Wild-type strain	**-**	Steven Berk
ETSU-22	Wild-type strain	**+**	Steven Berk
ETSU-25	Wild-type strain	**+**	Steven Berk
ETSU-26	Wild-type strain	**+**	Steven Berk
ETSU-W-1	Wild-type strain	**+**	Steven Berk
FIN2265	Wild-type strain	**-**	Merja Helminen
FIN2284	Wild-type strain	**-**	Merja Helminen
FIN2341	Wild-type strain	**+**	Merja Helminen
FIN2344	Wild-type strain	**-**	Merja Helminen
FIN2404	Wild-type strain	**-**	Merja Helminen
FIN2405	Wild-type strain	**-**	Merja Helminen
FIN2406	Wild-type strain	**-**	Merja Helminen
FIN2421	Wild-type strain	**-**	Merja Helminen
FIN3423	Wild-type strain	**-**	Merja Helminen
FIN6467	Wild-type strain	**-**	Merja Helminen
FR2213	Wild-type strain	**-**	Richard Wallace
FR2336	Wild-type strain	**-**	Richard Wallace
FR3227	Wild-type strain	**-**	Richard Wallace
4223	Wild-type strain	**-**	[56]
7169	Wild-type strain	**-**	[57]
A221	Wild-type strain	**-**	Richard Wallace
A225	Wild-type strain	**-**	Richard Wallace
B59504	Wild-type strain	**-**	David Goldblatt
B59911	Wild-type strain	**-**	David Goldblatt
C2	Wild-type strain	**-**	[58]
C9	Wild-type strain	**-**	[58]
MC317	Wild-type strain	**-**	[59]
O46E	Wild-type strain	**-**	[10]
P44	Wild-type strain	**-**	[58]
			
**Plasmids**			
pLQ510	Plasmid from *M. catarrhalis *E22	**+**	[24]
pLQ510.*mcbB::kan*	pLQ510 with a *kan *cartridge inserted within the *mcbB *ORF	**-**	This study
pLQ510.*mcbC::kan*	pLQ510 with a *kan *cartridge inserted within the *mcbC *ORF	**-**	This study
pWW115	Cloning vector for *M. catarrhalis*, spectinomycin-resistant	**-**	[52]
pAA111	pWW115 expressing a cloned His-tagged McbC protein	**+**	This study
pAA113	pWW15 expressing the wild-type McbI protein	**-**	This study

The *mcbABCI *locus in both *M. catarrhalis *O12E and V1120 was amplified by PCR and subjected to nucleotide sequence analysis. Comparison of the nucleotide sequence of the *mcbA *and *mcbB *ORFs from pLQ510 with the nucleotide sequence of the corresponding ORFs in O12E and V1120 showed at least 97% identity. Similarly, the deduced amino acid sequences for each of the three predicted McbA and McbB proteins were at least 98% identical. The proteins predicted to be encoded by the *mcbC *ORF in both pLQ510 and in the V1120 chromosome differed by only one amino acid (Figure 2A). However, the protein encoded by the *mcbC *ORF in strain O12E was shorter by 24 aa than that encoded by the *mcbC *ORFs in pLQ510 and V1120; this difference resulted from a change in the predicted translational initiation codon in strain O12E (data not shown). The remaining 77 aa in the O12E McbC protein differed by two residues from aa 25-101 in the pLQ510 McbC protein and by one residue from aa 25-101 in the V1120 McbC proteins (Figure 2A). The proteins encoded by the *mcbI *ORFs from pLQ510, O12E, and V1120 were identical.

### Construction of in-frame deletion mutations in the *mcb *locus in strain O12E

**Figure 5:**
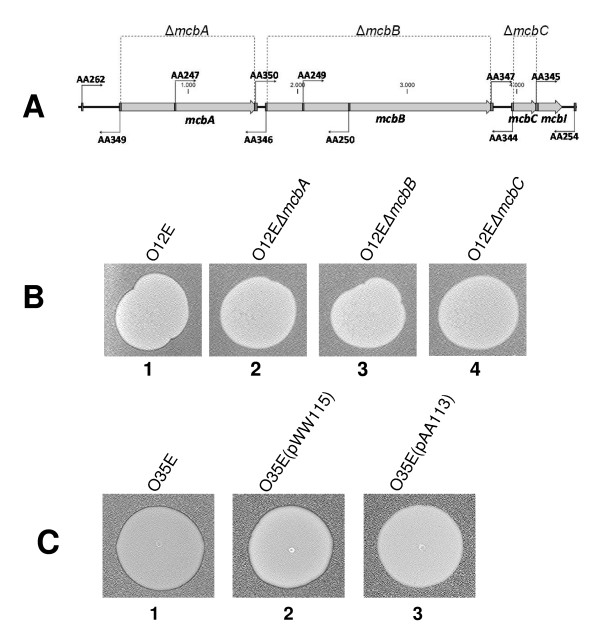
**Analysis of mutant and recombinant *M. catarrhalis *strains**. (A) Schematic showing the *mcbABCI *locus in the O12E chromosome and the position of the oligonucleotide primers used to construct the three different in-frame deletion mutations in this locus. The extent of the deletion in each ORF is indicated. (B) Bacteriocin production assay using O35E as the indicator strain together with the following test strains: panel 1, O12E; panel 2, O12EΔ*mcbA*; panel 3, O12EΔ*mcbB*; panel 4, O12EΔ*mcbC*. Panel C, Use of recombinant *M. catarrhalis *strains to demonstrate that expression of McbI in O35E confers protection against killing by strain O12E. *M. catarrhalis *O12E was used as the test strain in a bacteriocin production assay with three different *M. catarrhalis *strains as the indicator. Panels: A, O35E wild-type; B, O35E(pWW115) [vector-only control]; C, O35E(pAA113) [expressing McbI].

The O12EΔ*mcbA *mutant was constructed as described in Materials and Methods, such that McbA amino acids 8-411 are missing. PCR amplicons derived from the mutated plasmids pLQ510.*mcbB::kan *and pLQ510.*mcbC::kan *were used to transform O12E to obtain kanamycin-resistant *mcbB *and *mcbC *mutants. These kanamycin-resistant transformants were then used as targets for transformation by PCR amplicons that contained in-frame deletions within the *mcbB *and *mcbC *ORFs as described in Materials and Methods (Figure 5A). Strain O12EΔ*mcbB *has McbB amino acids 8-685 deleted, whereas strain O12EΔ*mcbC *has McbC amino acids 3-68 deleted (Figure 5A). In contrast to the parent strain O12E (Figure 5B, panel 1), each of these three mutants (Figure 5B, panels 2-4) was unable to kill strain O35E.

### The *mcbI *gene encodes an immunity factor

To determine whether the *mcbI *gene encoded an immunity factor, the *mcbI *gene from *M. catarrhalis *O12E was cloned into the plasmid vector pWW115 to obtain pAA113. A recombinant *M. catarrhalis *O35E strain containing pAA113 with the cloned *mcbI *gene (Figure 5C, panel 3) was resistant to killing by strain O12E. In contrast, both O35E (Figure 5C, panel 1) and O35E containing the empty vector pWW115 (Figure 5C, panel 2) were killed by strain O12E.

### Cloning and expression of the *mcbC *gene

**Figure 6:**
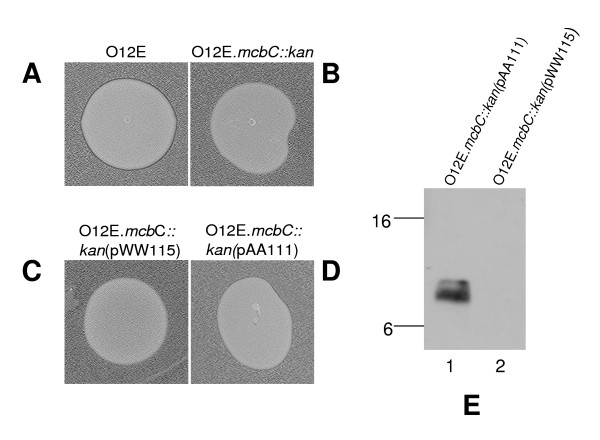
**Expression of the His-tagged *mcbC *gene product**. Killing of strain O35E by (A) wild-type O12E, (B) O12E.*mcbC::kan*; (C) O12E.*mcbC::kan*(pWW115); (D) O12E.*mcbC::kan*(pAA111). (E) Western blot-based detection of His-tagged McbC protein purified from spent culture supernatant fluid from (lane 1) *M. catarrhalis *O12E.*mcbC::kan*(pAA111) and (lane 2) *M. catarrhalis *O12E.*mcbC::kan*(pWW115) (negative control). A His-tag specific antibody was used as the primary antibody for Western blot analysis. Molecular weight position markers (in kDa) are present on the left side of this panel.

The *M. catarrhalis *O12E *mcbC *gene was cloned into pWW115 and modified such that the encoded McbC protein contained six histidine residues at its C-terminus (as described in Material and Methods). When expressed in the O12E.*mcbC::kan *mutant, the presence of this His-tagged McbC protein allowed killing of strain O35E (Figure 6D), although the degree of killing appeared to be slightly less than that obtained with the wild-type O12E strain (Figure 6A). In contrast, neither the O12E.*mcbC::kan *mutant (Figure 6B) nor this same mutant containing only the pWW115 vector (Figure 6C) killed O35E. Analysis of the purified His-tagged McbC protein showed that it migrated in SDS-PAGE (Figure 6E, lane 1) in a manner consistent with its calculated molecular weight of ~7,600 (calculated for the fusion protein after cleavage of the predicted leader sequence). This purified His-tagged McbC protein did not kill O35E (data not shown).

### Competitive growth experiments

Two different sets of co-culture experiments were performed to determine whether expression of the McbC bacteriocin would confer a growth advantage on a *M. catarrhalis *strain containing the *mcbABCI *locus. In the first, the bacteriocin-producing, streptomycin-resistant strain O12E-Sm^r ^and the spectinomycin-resistant, bacteriocin-sensitive mutant O35EΔ*mapA *[34] were mixed at a ratio of approximately 1:1 and grown in broth for 18 h. At the end of this growth period, O12E-Sm^r ^was the vastly predominant member (avg. 98.5%) of this culture. In a second set of experiments, O12E-Sm^r ^was co-cultured (starting inoculum ratio of 1:1) with either O35E containing the pWW115 vector or the recombinant plasmid pAA113 containing the wild-type O12E *mcbI *gene encoding the immunity factor. When O12E-Sm^r ^was grown overnight with O35E(pWW115), the bacteriocin-producing strain became predominant (avg. 99.76%) in the culture. In contrast, when the O35E strain expressed the *mcbI *gene product from a multi-copy plasmid, this recombinant strain persisted in the presence of the bacteriocin-producing strain such that *M. catarrhalis *O35E(pAA113) cells represented 76.9% of the total cells in the culture. It should be noted that, when all four of these strains were cultured independently in broth for 7-8 h, the O12E-Sm^r ^strain was shown to grow at approximately the same rate and to approximately the same extent as the other three strains (data not shown).

## Discussion

Bacteriocins are proteins and peptides that are ribosomally synthesized by many bacterial species and which usually have bactericidal activity against the same species or closely related bacteria. Bacteriocins range in size from the relatively large colicins (~60 kDa) synthesized by some *E. coli *strains to the very small (< 5 kDa) microcins [for reviews see [22,30,35]]. A significant number of bacteriocins, and especially those produced by lactic acid bacteria, have been studied for their potential to be used in food preservation [36]. The bacteriocins produced by the lactic acid bacteria are divided into two general classes. Class I bacteriocins undergo post-translational modification whereas class II microcins do not. These class II bacteriocins also have a characteristic leader sequence containing a double-glycine (GG) motif which is cleaved on the C-terminal side to release the mature bacteriocin [for a review see [35]].

In this study, we report the identification of a bacteriocin produced by *M. catarrhalis*. Despite the fact that the amino acid sequence of the mature McbC peptide did not show any significant homology to known bacteriocins, the sequence of the first 15 amino acids of the *M. catarrhalis *O12E McbC protein shows a high degree of conservation with leader peptides of proven and hypothetical class II bacteriocins from other bacteria (Figure 2B). The predicted McbC proteins encoded by the pLQ510 plasmid (in *M. catarrhalis *strain E22) and *M. catarrhalis *strain V1120, however, were both longer than the predicted O12E McbC protein, containing an additional 24 aa at the N-terminus. Because all three of these strains expressed killing activity against O35E, it appears that the shorter version of the McbC protein is functional with respect to bactericidal activity. Examination of the nucleotide sequence of the region preceding the two possible McbC translation initiation codons in both pLQ510 and V1120 indicated that the better predicted Shine-Dalgarno site was located immediately upstream of the second ATG (data not shown); this is the same ATG predicted to be the translation initiation codon for the O12E *mcbC *ORF.

Export of class II bacteriocins involves both an ATP-binding cassette (ABC) transporter and an accessory protein belonging to the membrane-fusion protein family [30]. The former protein also possesses proteolytic activity in an N-terminal domain [37] which belongs to the C39 peptidase superfamily [for a review see [31]]. The genes encoding both of these membrane-bound proteins are frequently located together with the ORFs encoding the bacteriocin and the host immunity factor [38]. Reverse transcriptase-PCR analysis of the locus in pLQ510 containing the gene encoding the McbC bacteriocin (Figure 3) indicated that it is located in an operon where it is preceded by the *mcbA *and *mcbB *genes which encode a predicted accessory protein (McbA) belonging to the membrane-fusion family and an ABC transporter (McbB), respectively. A previous BLAST-based survey identified the protein encoded by *mcbB *as an ABC transporter, although no more detailed analysis of this protein was provided by these authors [30].

The 3'-end of the *mcbC *gene is overlapped by the 5'-end of another ORF which encodes the immunity factor McbI. Similar ORF overlaps, described previously for other bacteriocin-producing systems, would allow tight co-regulation of the production of the bacteriocin and its cognate immunity factor [39,40]. The function of the McbI protein was deduced from an experiment in which the presence of the *mcbI *gene on a multi-copy plasmid protected the McbC-sensitive O35E strain from killing by the McbC-producing O12E strain (Figure 5C). The McbI protein contains only 74 amino acids and did not show a high degree of amino acid sequence homology to other immunity proteins, a result which is not unusual [39]. However, the predicted secondary structure of McbI showed the presence of four α-helices, a feature that is conserved among class IIa immunity proteins [35,41]. Precisely how the immunity protein confers protection against its cognate bacteriocin has been elucidated for at least one class II bacteriocin [42].

While the *mcbABCI *locus was first identified in the plasmid pLQ510, the ability to kill O35E was not restricted to the E22 strain carrying this plasmid. Instead, 12 of another 54 *M. catarrhalis *strains tested in the present study could kill O35E. Moreover, the presence of the bacteriocin locus in at least some of these other *M. catarrhalis *strains is apparently not dependent on the presence of an extrachromosomal element. Two *M. catarrhalis *strains (O12E and V1120) which were able to kill O35E also had the *mcbA*, *mcbB*, and *mcbC *genes located in their chromosome in the absence of any plasmids detectable by a basic plasmid isolation technique. In this regard, it is interesting to note that the original report describing the existence of pLQ510 in strain E22 indicated that some pLQ510 plasmid sequences were detected by Southern blot analysis in the chromosome of another *M. catarrhalis *strain that apparently lacked plasmids [24].

Efforts to obtain killing activity with filter-sterilized, spent culture supernatant fluids from a *M. catarrhalis *strain containing the *mcbABCI *locus were not successful (data not shown). It is interesting that the killing zone produced by the strains carrying the *mcbABCI *locus is very small (Figure 1C and Figure 4A). It is possible that the in vitro growth conditions used in this study were not optimal for bacteriocin production by *M. catarrhalis*, and that there may exist an environmental signal which will increase synthesis and release of this bacteriocin. Other bacteriocins can often be concentrated from spent culture supernatant fluids [43-45], and it is difficult to explain our inability to accomplish this with the McbC protein. Similarly, a purified, His-tagged McbC protein was not able to kill a sensitive strain in vitro (data not shown). Whether the quantity of purified McbC protein was insufficient, whether the purification procedure inactivated this fusion protein, or whether the His tag may have interfered with McbC bactericidal activity cannot be determined from the available data.

The true biological role of the McbC bacteriocin remains to be determined. Results presented in the present study suggest that the McbC protein likely has a relatively narrow range of activity, apparently being only able to kill *M. catarrhalis *strains that are lacking the *mcbABCI *locus. Expression of McbC might mediate some type of intraspecies competition in the nasopharynx, as has been described for the BlpMN bacteriocins of *Streptococcus pneumoniae *[46]. In addition, inactivation of a gene involved in bacteriocin production in *Neisseria meningitidis *was recently shown to adversely affect the ability of the mutant to colonize in a human nasal pharyngeal organ culture model [47].

In a preliminary effort to determine whether McbC might be able to kill other members of the normal flora of the human oropharynx and thereby facilitate colonization of the mucosa by *M. catarrhalis*, we performed growth inhibition experiments using two different α-hemolytic streptococci [*Streptococcus mitis *NS 51 (ATCC 49456) and the *Streptococcus sanguinis *type strain (ATCC 10556)] as the indicator strains. However, *M. catarrhalis *O12E had no detectable inhibitory effect on the growth of these two strains (data not shown). The limited spectrum of killing activity for McbC also raises the possibility that it might serve to lyse other *M. catarrhalis *strains that lack the *mcbABCI *locus, thereby making their DNA available for lateral gene transfer via transformation of the strain containing the *mcbABCI *operon. A similar mechanism has been described for how *Streptococcus mutans *might use its mutacin (bacteriocin) to acquire genes from closely related streptococcal species in vivo [48].

## Conclusion

Approximately 25% of the *M. catarrhalis *strains tested in this study produced a bacteriocin that could kill strains of this pathogen that lacked the *mcbABCI *locus. Expression of the gene products encoded by this locus conferred a competitive advantage in vitro over a strain that did not possess this set of genes. Whether this bacteriocin is expressed in vivo (i.e., in the human nasopharynx) remains to be determined, but production of this bacteriocin could facilitate lateral gene transfer among *M. catarrhalis *strains.

## Methods

### Bacterial strains, plasmids and growth conditions

Bacterial strains and plasmids used in this study are listed in Table 1. *Moraxella catarrhalis *strains were routinely grown in brain heart infusion (BHI) broth (Difco/Becton Dickinson, Sparks, MD) with aeration at 37°C, or on BHI solidified using 1.5% (wt/vol) agar. When appropriate, BHI was supplemented with kanamycin (15 μg/ml), streptomycin (100 μg/ml), or spectinomycin (15 μg/ml). BHI agar plates were incubated at 37°C in an atmosphere containing 95% air-5% CO_2_. Mueller-Hinton (MH) broth (Difco/Becton Disckinson) was used for some growth experiments involving co-culture of two different *M. catarrhalis *strains. *Streptococcus mitis *NS 51 (ATCC 49456) and the *Streptococcus sanguinis *type strain (ATCC 10556) were obtained from the American Type Culture Collection (Manassas, VA) and were grown on blood agar plates.

### Detection of bacteriocin production

*M. catarrhalis *strains were tested for bacteriocin production by growing both the test strain (i.e., the putative bacteriocin-producing strain) and the indicator strain (i.e., the putative bacteriocin-sensitive strain) separately in BHI broth overnight at 37°C. The cells of the indicator strain were collected by centrifugation and resuspended in a 5 ml portion of BHI to an OD_600 _= 0.25. The cells of the test strain were collected by centrifugation and resuspended in a 1 ml volume of BHI. A 250-μl portion of the suspension of the indicator strain was used to inoculate a flask containing 25 ml of molten BHI agar [0.8% (wt/vol) agar] at a temperature of 45°C. A 10-ml portion of the inoculated molten BHI agar was then spread over approximately 20 ml of solidified BHI agar in a Petri dish. The plates were allowed to solidify and then 10-μl portions of the test strain suspension were spotted on the surface of the agar. These plates were then incubated at 37°C overnight. Production of bacteriocin by the test strain and/or the susceptibility of the indicator strain were indicated by the presence of a small clear zone of growth inhibition around the test strain.

### PCR-based detection of the *mcb *locus

Chromosomal DNA was prepared from eight *M. catarrhalis *strains and used in PCR with the oligonucleotide primers AA247 (5'-TGCCATTGCCAAAGAGAC-3') and pLQ510-rp1 (5'-CACCATATGACAATCTATTAG-3'). AA247 was located in the *mcbA *ORF and pLQ510-rp1 was located in the *mcbC *ORF. Nucleotide sequences of the *mcbABCI *genes from *M. catarrhalis *strain O12E were deposited at GenBank and assigned the following accession numbers: *mcbA*, EU780917; *mcbB*, EU780918; *mcbC*, EU780919; *mcbI*, EU780920. The *mcbABCI *genes from *M. catarrhalis *strain V1120 were deposited at GenBank and assigned the following accession numbers: *mcbA*, EU755328; *mcbB*, EU755329; *mcbC*, EU755330; *mcbI*, EU755331.

### Inactivation of selected genes in pLQ510

The *mcbB *ORF was inactivated by ligating a kanamycin resistance cassette [49] into the BsiWI site within this ORF in pLQ510; the new plasmid was designated pLQ510.*mcbB::kan*. The *mcbC *ORF was inactivated by inserting a kanamycin resistance cassette into the HpaI site in this ORF; the new plasmid was designated pLQ510.*mcbC::kan*.

### Construction of deletion mutations in the chromosome of *M. catarrhalis *strain O12E

To construct an in-frame deletion in the *mcbA *gene, primers AA262 (5'- GAAGT AAATCGTCAGATGG-3') and AA349 (5'-AGGGCGGAATAGACTAGACAT-3') were used to amplify a DNA fragment containing the 345 nucleotides (nt) upstream of the *mcbA *ORF together with the first 21 nt of this ORF, using chromosomal DNA from strain O12E as a template. Primers AA350 (5'-AGTCTATTCCGCCCTCCGCT ATATAGT CTCACAGGTAAAATTTAA-3') and AA250 (5'-AAAACTGGCTGG GCAGATG-3') were used to amplify the last 30 nt of the *mcbA *ORF together with 855 nt of the downstream DNA. The resultant two PCR products were used as templates in overlapping extension PCR [50] using primers AA262 and AA250. The new PCR product was used in a plate transformation system [51] to transform *M. catarrhalis *strain O12E. Transformants were screened by colony-PCR using primers AA262 and AA251 (5'-AGATTGCTCACTCGTCCAC-3'); this latter primer binds downstream of AA250. One transformant shown to contain the desired deletion in the *mcbA *gene was designated O12EΔ*mcbA*.

For the construction of an in-frame deletion in the *mcbB *ORF, primers AA247 (5'-TGCCATTGCCAAAGAGAC-3') and AA346 (5'-AATATTCTTTAAAAAATC CAT-3') were used to amplify 830 nt upstream of the *mcbB *ORF together with the first 21 nt of the *mcbB *ORF using chromosomal DNA from strain O12E as the template. In addition, primers AA347 (5'-TTTTTAAAGAATATTAGCACTGATT GGGTACTGAACCTTGGTTAA-3') and AA254 (5'-GGGCTTTGGGCGGTA GGTTATTA-3') were used to amplify the last 30 nt of the *mcbB *ORF and 769 nt of the downstream region. Both PCR fragments were used as templates for an overlapping extension PCR using primers AA247 and AA254; the resultant amplicon was designated 247-254. Wild-type strain O12E was first transformed with a PCR amplicon obtained by using primers AA248 (5'-CTGTTGCCAAAACTGCTC-3') and AA252 (5'-GCACATTGTTCCACCCATTCA-3') with plasmid pLQ510.*mcbB::kan *as the template; this amplicon contained the *mcbB *gene and the inserted *kan *cartridge. One of the resultant kanamycin-resistant transformants (O12E.*mcbB::kan*) was subsequently transformed with the 247-254 amplicon. Transformants were screened for the loss of kanamycin resistance and one kanamycin-sensitive transformant was selected for further study and designated as O12EΔ*mcbB*.

To construct an in-frame deletion in the *mcbC *ORF, the same strategy was employed as was used for construction of the O12EΔ*mcbB *mutant. The primer pair AA249 (5'-TTAGACCC AAGTGCTGGAC-3') and AA344 (5'-ACGCATAATATATTCCTTT AT-3') and the primer pair AA345 (5'-GAATATATTATGCGTATTATGGTTG GAGTTACTAAAAAATGGTAA-3') and AA254 were used in the initial PCR reactions with O12E chromosomal DNA, and the final amplicon containing a deletion in the *mcbC *ORF was used to transform an O12E mutant which had a kanamycin resistance cassette in its *mcbC *ORF (i.e., O12E.*mcbC::kan*). One kanamycin-sensitive transformant was selected for further characterization and was designated O12EΔ*mcbC*. PCR and nucleotide sequence analysis were used to confirm that these three deletion mutations (i.e., in O12EΔ*mcbA*, O12EΔ*mcbB*, and O12EΔ*mcbC*) were in-frame.

### Reverse transcriptase-PCR

Possible transcriptional linkage among the ORFs in the *mcb *locus in pLQ510 was assessed by the use of reverse transcriptase-PCR. Total RNA was isolated from mid-logarithmic phase cells of *M. catarrhalis *E22 by using the RNeasy midi kit (Qiagen). RNA samples were treated with DNase I (Message Clean Kit, GenHunter Corp, Nashville, TN) to remove any DNA contamination. To amplify the region between the *mcbA *and *mcbB *ORFs, primers *mcb *A/B fw (5'-TAGCAGTTGGCATGACC TTG-3') and *mcb *A/B rv (5'-AGCAAGACAGGCTAGACCACA-3') were used. For the region between *mcbB *and *mcbC*, primers *mcb *B/C fw (5'-AGAGCGCTGATTG GGTACTG-3'), and *mcb *B/C rv (5'-CAT GCCATTGACTGACCAAC-3'), were used, and for the region between *mcbC *and *mcbI*, primers *mcb *C/I fw (5'-TCCTA ATAGATTGTCATATGGTGGTT-3') and *mcb *C/I rv (5'-CAAAACG TGCACA ATTAGGG-3') were used. The reverse transcriptase reaction was carried out using MultiScribe reverse transcriptase (Applied Biosystems, Foster City, CA) followed by PCR amplification. In addition, the reaction was also performed using either chromosomal DNA alone as the template or with the RNA template in the absence of reverse transcriptase.

### Construction of a plasmid encoding a His-tagged McbC protein

In order to construct a plasmid that expressed the McbC protein with a C-terminal His-tag, the primer pair AA357 (5'-TGGGATCCGGTACTATTTAATGTACTAA GATTTT-3') (BamHI site underlined) and AA359 (5'-GTGGTGGTGGTGGTGGTG CCATTTTTTAGTAACTCCAACCATAAT-3') and the primer pair AA358 (5'-CACCACCACCACCACCACTAAAGACAATAGGTTTAGCATGGATAT-3') (*mcbC *translational stop codon underlined) and AA354 (5'-GGTTGAGCTCCCA TTTAAGTGATTTTGTTATATCAAT-3') (SacI site underlined) were used to generate two PCR products using O12E chromosomal DNA as the template. The resultant two PCR products were used as templates for an overlapping extension PCR involving primers AA357 and AA354. The final PCR amplicon was then digested with both BamHI and SacI and ligated into pWW115 [52] that had been digested with these same restriction enzymes. The ligation mixture was used to transform O12E.*mcbC::kan*. A plasmid isolated from a spectinomycin-resistant colony and which expressed the His-tagged McbC protein was designated pAA111. Plasmid pWW115 was used to transform *M. catarrhalis *O12E.*mcbC::kan *to provide a negative control.

### Purification and detection of the His-tagged McbC protein

*M. catarrhalis *O12E.*mcbC::kan*(pWW115) and *M. catarrhalis *O12E.*mcbC::kan*(pAA111) were grown independently in 1 L BHI overnight at 37°C with shaking. The cultures were subjected to centrifugation to pellet the bacterial cells and the supernatant fluid was filter-sterilized. Two columns each containing 1.5 mL of NiNTA agarose beads (Qiagen, Valencia, CA) were washed with washing buffer (50 mM NaH_2_PO_4_, 200 mM NaCl, 5 mM imidazole [pH 7.9]). The culture supernatant fluids were passed through the columns twice after which the columns were washed with washing buffer again. The His-tagged protein was eluted using elution buffer (50 mM NaH_2_PO_4_, 200 mM NaCl, 200 mM imidazole [pH 7.9]). Selected fractions were pooled and dialyzed against PBS. SDS-digestion buffer was added to a final concentration of 1× to each sample. For Western blot analysis, proteins were resolved by SDS-PAGE using 15% (wt/vol) polyacrylamide separating gels and transferred to polyvinylidene difluoride membranes. The anti-His tag antibody HIS.H8 (Millipore, Temecula, CA) was used at a dilution of 1:2,000 in PBS-Tween containing 3% (wt/vol) dried milk and incubated with the membrane for 2 h at room temperature. Horseradish peroxidase-conjugated goat anti-mouse antibody (Jackson Immunoresearch, West Grove, PA) was used as the secondary antibody. The antigen-antibody complexes were detected by using Western Lightning Chemiluminescence Reagent Plus (New England Nuclear, Boston, MA).

### Construction of a plasmid containing the *mcbI *gene

Primers AA353 (5'-ATGGATCCGAAAACTCATTGGGGAGATAGAGGGAT-3') (BamHI site underlined) and AA378 (5'-TTGTGAGCTCGCTCGGATTTGCTATTATTGA-3') (SacI site underlined) were used to PCR-amplify a 288-bp fragment containing the *mcbI *gene from *M. catarrhalis *O12E chromosomal DNA. The resultant PCR product was digested with both BamHI and SacI and ligated into pWW115 which had been digested with the same two restriction enzymes. This ligation mixture was used to transform *M. catarrhalis *strain O35E and transformants were selected for spectinomycin resistance. The plasmid from one of these transformants was designated pAA113.

### Competitive index-based broth growth experiments

A streptomycin-resistant mutant of the wild-type strain O12E (O12E-Sm^r^) [53] and the spectinomycin-resistant recombinant strains O35E(pWW115) and O35E(pAA113) were grown separately in MH broth to a density of approximately 10^8 ^CFU/ml. Equal volumes of O12E-Sm^r^and the individual recombinant O35E strains were mixed in a 1:1 ratio. Serial dilutions of this mixture were plated on BHI agar plates containing the appropriate antibiotic to determine the relative percentages of each strain in the input mixture. Either a 1 ml or a 0.5 ml portion of the mixture was used to inoculate either 250 ml or 125 ml of MH broth, respectively, which was then allowed to grow overnight at 37°C with aeration. The cells were harvested after 18 h of growth, serially diluted, and plated on agar-based media containing the appropriate antibiotic to determine the relative percentage of each strain in the output mixture. A second set of competition experiments involving O12E-Sm^r ^and the spectinomycin-resistant mutant O35EΔ*mapA *[34] was performed similarly. Each co-culture experiment was done three times independently; the data are the mean of the three experiments.

## Authors' contributions

ASA, LL, and EJH conceived of the study and participated in its design. ASA and LL designed, constructed, and characterized mutants. JLS designed and executed the competition experiments, and performed additional mutant analyses. TCH and WL designed and executed RT-PCR experiments. CAB performed analysis of protein structure and provided bioinformatics. ASA and EJH drafted the manuscript. All authors read and approved the final manuscript.
